# The Potential for Transmission of BCG from Orally Vaccinated White-Tailed Deer (*Odocoileus virginianus*) to Cattle (*Bos taurus*) through a Contaminated Environment: Experimental Findings

**DOI:** 10.1371/journal.pone.0060257

**Published:** 2013-04-02

**Authors:** Pauline Nol, Jack C. Rhyan, Suelee Robbe-Austerman, Matt P. McCollum, Tara D. Rigg, Nadia T. Saklou, Mo D. Salman

**Affiliations:** 1 United States Department of Agriculture, Animal and Plant Health Inspection Service, Veterinary Services, National Wildlife Research Center, Fort Collins, Colorado, United States of America; 2 United States Department of Agriculture, Animal and Plant Health Inspection Service, Veterinary Services, National Veterinary Services Laboratories, Ames, Iowa, United States of America; 3 United States Department of Agriculture, Animal and Plant Health Inspection Service, Wildlife Services, National Wildlife Research Center, Fort Collins, Colorado, United States of America; 4 Animal Population Health Institute, Department of Clinical Sciences, College of Veterinary Medicine and Biomedical Sciences, Colorado State University, Fort Collins, Colorado, United States of America; Glaxo Smith Kline, Denmark

## Abstract

White-tailed deer (*Odocoileus virginianus*) experimentally infected with a virulent strain of *Mycobacterium bovis* have been shown to transmit the bacterium to other deer and cattle (*Bos taurus*) by sharing of pen waste and feed. The risk of transmission of *M. bovis* bacille Calmette-Guerin (BCG) vaccine from orally vaccinated white-tailed deer to other deer and cattle, however, is not well understood. In order to evaluate this risk, we orally vaccinated 14 white-tailed deer with 1×10^9^ colony forming units BCG in lipid-formulated baits and housed them with nine non-vaccinated deer. Each day we exposed the same seven naïve cattle to pen space utilized by the deer to look for transmission between the two species. Before vaccination and every 60 days until the end of the study, we performed tuberculin skin testing on deer and cattle, as well as interferon-gamma testing in cattle, to detect cellular immune response to BCG exposure. At approximately 27 weeks all cattle and deer were euthanized and necropsied. None of the cattle converted on either caudal fold, comparative cervical tests, or interferon-gamma assay. None of the cattle were culture positive for BCG. Although there was immunological evidence that BCG transmission occurred from deer to deer, we were unable to detect immunological or microbiological evidence of transmission to cattle. This study suggests that the risk is likely to be low that BCG-vaccinated white-tailed deer would cause domestic cattle to react to the tuberculin skin test or interferon-gamma test through exposure to a BCG-contaminated environment.

## Introduction

Bovine tuberculosis, caused by *Mycobacterium bovis* infection, poses a serious continual threat to the health and economic well-being of both livestock and humans worldwide. In several countries, a major obstacle preventing the eradication of this disease is its presence in wildlife populations [1]. An important example of a wildlife reservoir for *M. bovis* is the wild white-tailed deer (*Odocoileus virginianus*; WTD) population in northeastern lower peninsular Michigan, United States of America (USA), where outbreaks in cattle (*Bos taurus*) and other domestic stock continue to occur [2], [3]. One proposed control strategy for bovine tuberculosis in Michigan is the implementation of an oral vaccination program for WTD. *Mycobacterium bovis* bacille Calmette-Guerin (BCG) is a live bacterial vaccine and the only tuberculosis vaccine available for human use. This vaccine is effective in protecting WTD from disease caused by *M. bovis* infection [4], [5], [6]. This vaccine would therefore be a likely candidate for a WTD vaccination program in Michigan and elsewhere.

A potential problem that could arise as a consequence of oral BCG vaccination of wildlife would be the accidental exposure of domestic cattle to BCG. Exposure can come in the form of unintended ingestion of vaccine, and also direct or indirect transmission from vaccinated deer. Both situations could result in cattle responding to tuberculin skin testing or interferon-gamma testing, the primary diagnostic tools based on measuring cellular immune responses, used to test for *M. bovis-*infected cattle.

White-tailed deer experimentally infected with field strain *M. bovis* have been shown to transmit this bacterium to deer and cattle by sharing pen waste and feed [7]. In a study where WTD were parenterally vaccinated with BCG, however, cattle that were exposed to their pen waste and feed did not react to skin testing or interferon gamma testing, nor were any of their tissues positive for BCG [8]. As wild WTD would be most likely vaccinated via the oral route, evidence is still needed to determine whether WTD orally vaccinated with BCG are likely to transmit BCG via shared feed or bedding to cattle. The aims of this study were to document evidence through skin testing and interferon-gamma measurement of transmission of BCG from orally-vaccinated WTD to domestic cattle exposed to a contaminated environment, and document transmission between vaccinated and non-vaccinated WTD sharing the same pen.

## Materials and Methods

This study was carried out under a protocol approved by the Institutional Animal Care and Use Committee of Colorado State University, Fort Collins, Colorado, USA (Protocol #: 08-128A-01**).**

Twenty-three yearling and adult white-tailed deer were acquired from two bovine tuberculosis-free captive herds: The adult WTD (n = 7) were obtained from a research herd in Washington, USA and brought to the Animal Population Health Institute’s Wildlife Research Facility (WRF) at Colorado State University. The yearling WTD (n = 16) were obtained as neonates from Missouri, USA and were hand-raised at the WRF. Seven yearling Jersey and Jersey-Holstein cross steers were obtained as calves from a bovine tuberculosis-free dairy in Meade, Colorado, USA and also raised at the WRF. The deer were randomly placed in one of two groups (Vaccinates; n = 14 and Non-vaccinates; n = 9) by using the RANDbetween function in Excel (Microsoft, Redmond, Washington, USA). Those in the vaccinate group were given 1×10^9^ colony forming units (cfu) *M. bovis* BCG Danish 1331 incorporated into a lipid bait. Each vaccinate was fed a single bait by hand. These baits were manufactured by and purchased from Otago Innovations Ltd. (Dunnedin, New Zealand). The nine non-vaccinated deer were not offered any treatment and remained in direct contact with vaccinated deer by sharing the same pen. The steers were housed adjacent to the deer but were never allowed direct contact as the pens were divided by solids metal, 8-foot high panels. Every twenty-four hours the deer and steers were moved to the pen that the other species had occupied the previous day. On a daily basis, overall health and behavior of the animals were noted and any abnormalities recorded.

Before vaccination, and at 9, 18, and 25 weeks post-vaccination, all the animals were tested for exposure to BCG via tuberculin skin testing according to USDA guidelines [9]. All deer were tested using the comparative cervical skin test (CCT). The steers were first tested using the caudal fold test (CFT) followed by the CCT. For the CFT, 0.1 ml *M. bovis* PPD (1.0 mg/ml; National Veterinary Services Laboratories, Ames, Iowa, USA; NVSL) was injected intradermally in the caudal fold area. Seventy-two hours later, the injection site was evaluated visually and by palpation for swelling and induration. After reading of the CFT, the CCT was administered according to USDA guidelines. In both species, two sites on the lateral neck were clipped and cleaned with isopropanol. Skin thickness was measured prior to injection to the nearest millimeter using calipers. 0.1 ml *M. bovis* PPD (1.0 mg/ml) and 0.1 ml (0.4 mg/ml) *M. avium* PPD (NVSL) were injected intradermally into separate clipped sites on the neck. Seventy-two hours later, injection sites were evaluated visually, by palpation, and by skin thickness. Change in skin thickness due to swelling or induration was calculated by subtracting the pre-injection skin thickness from the post-injection skin thickness that was obtained 72 h after injection. Deer and cattle were classified as reactors, non-reactors, or suspects according to USDA guidelines using Veterinary Services Form 6-22D [9]. For analyses, deer that were suspect skin test responders were classified as non-reactors. At the above time points except the final, the cattle were also tested using an interferon gamma assay (Bovigam™, Prionics, Schlieren, Switzerland) according to manufacturer’s instructions with an additional well composed of a positive control using 20 µg/ml pokeweed mitogen (Sigma Aldrich Co., St. Louis, Missouri, USA).

At the 27^th^ week post-vaccination the deer were euthanized and necropsied. Between 28 and 32 weeks post-vaccination, the steers were euthanized and necropsied. The following tissues were collected and stored at −70 C until transport to NVSL for culture of *Mycobacterium* spp., including non-tuberculous *Mycobacterium* spp. (NTM), as described by Palmer et al. (2010a): palatine tonsil, pharyngeal tonsil, parotid lymph nodes (ln) submandibular ln, medial retropharyngeal ln, mediastinal ln, tracheobronchial ln, hepatic ln, mesenteric ln, iliac ln, superficial inguinal ln, and popliteal ln. Tissue samples were also collected in 10% buffered formalin for as-needed histopathological inspection. Lymph nodes were pooled for culture as follows: head pool (medial retropharyngeal, parotid, and mandibular ln); oral lymphoid tissue pool (palatine tonsil, pharyngeal tonsil); thoracic pool (tracheobronchial and mediastinal ln); abdominal pool (mesenteric and hepatic ln), and caudal pool (iliac, superficial inguinal, and popliteal ln). Tissues that grew *Mycobacterium* spp. were prepared histologically and stained with both hematoxylin and eosin and Ziehl-Neelsen stains.

Vaccinates and non-vaccinates were compared based on skin test responses at each time point. Deer that grew NTM after necropsy were compared with deer that did not grow NTM based on final skin test responses. Both comparisons were made using 2×2, 2-sided Fisher’s Exact Test (ProcFREQ; SAS 9.1, SAS Institute, Cary, North Carolina, USA). Due to the exploratory nature of this experiment and relatively small sample sizes, differences determined to have a P value ≤0.1 were considered significant.

## Results

**Figure 1:**
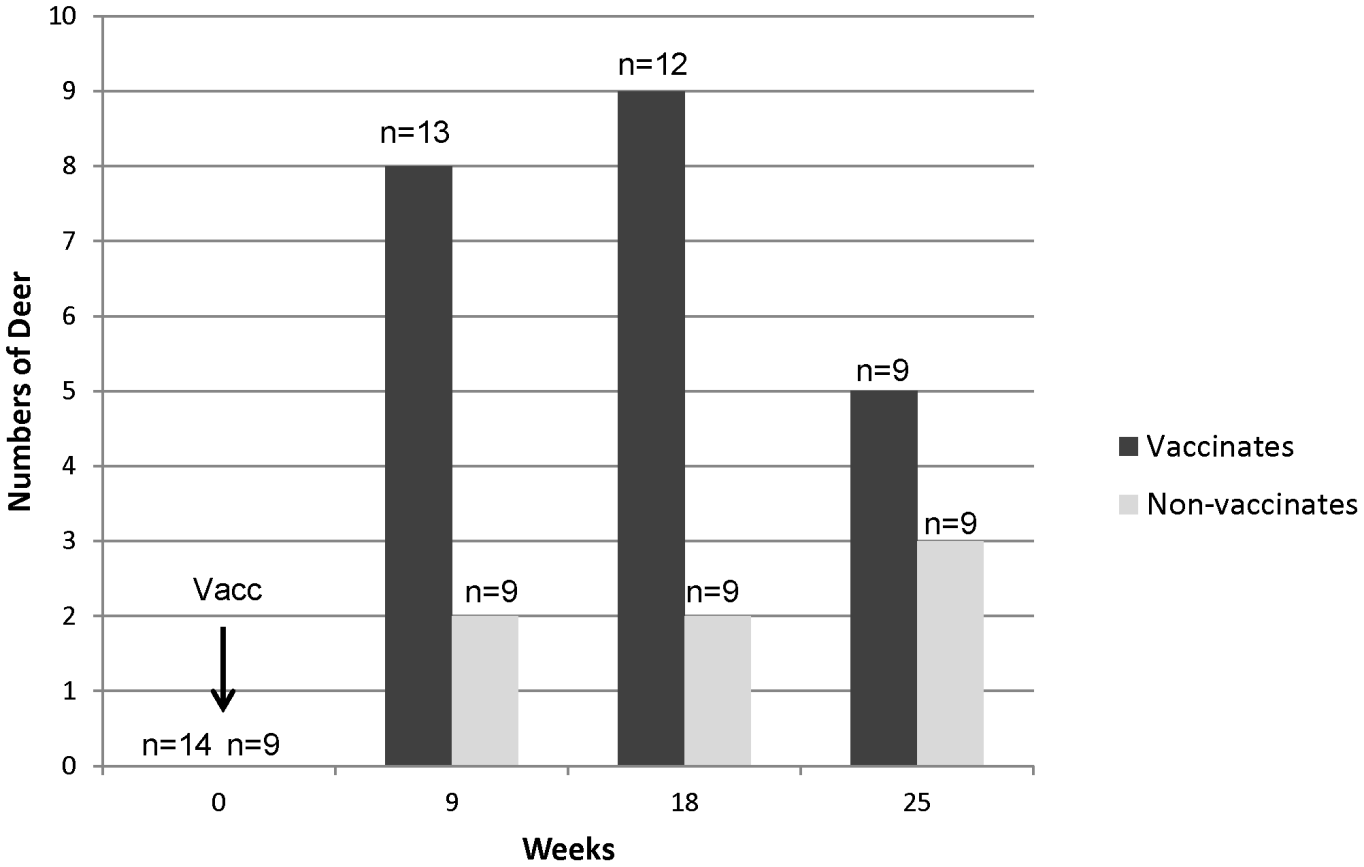
Numbers of CCT reactor white-tailed deer at four time points. Numbers of vaccinated and non-vaccinated white-tailed deer of total tested that were comparative cervical skin test reactors as measured at four time points.

Five vaccinated deer died before termination of the study at 5 (n = 1), 12 (n = 1), and 22 (n = 3) weeks post-vaccination. Causes of death included pneumonia (n−1), bacterial meningitis (n = 1), pericarditis and pneumonia (n = 1), bilateral nephrosis (n = 1), and unknown (n = 1). Ante-mortem test results were included when available, and tissues were collected for histology and culture at the time of death for the deer that died prematurely. Of the 13 vaccinated deer tested after vaccination, 11 were CCT reactors at some time point. Of the 9 in-contact, non-vaccinated deer, 5 became reactors on CCT at some time point after vaccination (Table 1; Figure 1). Prior to vaccination, none of the deer in either group were reactors on skin test, although one of the non-vaccinates was a suspect. At 9 and 18 weeks post-vaccination, the vaccinated group had a significantly greater proportion of reactors on skin test than did the non-vaccinates (p = 0.1 and p = 0.03 respectively). By 25 weeks, however, the proportion of reactors was not significantly different between the groups (p = 0.6). Skin test results were not universally consistent from one time point to the next, therefore animals did not necessarily remain suspects or reactors after initially becoming suspects or reactors (Table 1). None of the steers ever became reactors on CFT, CCT, or interferon gamma assay throughout the experiment.

**Table 1:** Comparative cervical test (CCT) results in individual deer at each time point and outcomes of tissue culture at necropsy.

		CCT*					
		Weeks Post-vaccination					
Animal ID	Group	0	9	18	25	Mycobacterial Culture	Tissue Pool
2	Non-vaccinate	N	R	N	R	*M. avium hominisuis*	Thoracic
3	Non-vaccinate	N	N	R	N	–	
9	Non-vaccinate	S	N	R	R	–	
17	Non-vaccinate	S	S	N	R	–	
22	Non-vaccinate	N	R	S	S	*M. avium hominisuis*	Thoracic
24	Non-vaccinate	N	N	N	N	*M. avium hominisuis*	
26	Non-vaccinate	N	N	S	N	*M. setense*	Head
Y25	Non-vaccinate	N	N	N	S	–	
Y98	Non-vaccinate	N	N	S	S	*M. avium hominisuis*	Thoracic
1	Vaccinate	N	S	S	R	–	
4	Vaccinate	N	R	R	N	–	
5	Vaccinate	N	R	R	S	*M. setense*	Head
12	Vaccinate	N	N	R	R	*M. avium* complex	Thoracic
18	Vaccinate	N	R	R	R	–	
25	Vaccinate	N	R	R	R	–	
28	Vaccinate	S	R	.	.	*M. avium hominisuis*	Thoracic/Abdominal
29	Vaccinate	N	S	N	.	–	
30	Vaccinate	N	N	R	.	–	
Y21	Vaccinate	N	F	R	S	–	
Y22	Vaccinate	N	N	N	N	–	
Y92	Vaccinate	N	.	.	.	–	
Y93	Vaccinate	N	R	R	.	–	
Y95	Vaccinate	N	R	R	R	–	

* N = negative; S = suspect; R = reactor.

None of the WTD or steers was culture positive for *M. bovis* or BCG. Three species of NTM were obtained from lymph node pools from WTD (*M. avium* Complex (n = 1), *M. avium hominisuis* (n = 5), and *M. setense* (n = 2)) and *M. avium hominisuis* was obtained from a thoracic pool from one steer. Vaccination status and corresponding CCT data from the NTM-positive deer are reported in Table 1. There was no significant difference between deer that were NTM-positive and NTM-negative in terms of skin test outcome, regardless of vaccine status (p = 0.7). The post-power calculation for these non-significant results is estimated to be 67%.

Hematoxylin and eosin and Ziehl-Neelsen-stained sections of thoracic lymph nodes (mediastinal and tracheobronchial) from six deer, head lymph nodes from two deer (submandibular, medial retropharyngeal, and parotid), and thoracic lymph nodes from one steer found to be culture-positive for NTM were examined. There was no evidence of lesions consistent with mycobacterial infection in these tissues and no acid fast organisms were observed.

## Discussion

Based on our skin test and interferon gamma assay data, we found no immunological evidence of BCG transmission between white-tailed deer vaccinated orally with 1×10^9^ cfu BCG and cattle exposed to feed and pen waste from vaccinated deer. We did find immunological evidence that BCG may have been transmitted from vaccinated deer to non-vaccinated pen mates; however, we were unable to determine actual presence of BCG infection in the tissues of any deer. These findings are consistent with those of Palmer and others [8], who conducted a very similar study vaccinating white-tailed deer subcutaneously with BCG and found no evidence of transmission to cattle. As also reported by Palmer et al [8], we observed development of reactors on CCT in in-contact pen mates, suggesting that some BCG transmission may have occurred between vaccinated and non-vaccinated deer. In contrast, a study examining virulent *M. bovis* did demonstrate transmission to cattle from infected deer through a contaminated environment [7], [10], [11]. The power of the findings is limited mainly due to the small sample size. Increase the sample size was prohibited due to the limited resources including space for these types of experiments.

The comparative cervical skin test results from this study indicate that deer exposed to BCG through oral vaccination can demonstrate responses on that test. That the majority of vaccinates and nearly half the in-contact deer reacted to skin test is consistent with observations from parenterally vaccinated deer but not so with findings of several oral BCG studies done in cattle. Palmer and others [8], [10] found that 11 of 15 deer subcutaneously vaccinated with BCG were CCT positive at 180 days post-vaccination, as were four of eight in-contact non-vaccinates. In two cattle studies, Buddle and others [12], [13] found that in animals vaccinated orally with 1×10^9^ cfu BCG via lipid-formulated bait, only one of 10 animals at 11 weeks post-vaccination and two of 10 animals at 17 weeks post-vaccination became CCT reactors. These findings in white-tailed deer may reflect unique immune characteristics regarding response to exposure to BCG.

We were unable to detect presence of BCG in any tissues collected from cattle or deer in our study. These findings may suggest that transmission of BCG did not occur between deer and cattle in this study. Although we had evidence through skin test data that there may have been transmission of BCG between vaccinated and non-vaccinated deer, we could not confirm this transmission through culture data. Previous studies have shown that BCG can persist in WTD lymph nodes for six to nine months after parenteral vaccination but only for up to three months after oral vaccination [5], [8]. Palmer et al. [8] reported only one of ten in-contact non-vaccinates having cultured positive for BCG in both head and thoracic lymph nodes six months after parenteral vaccination of pen mates with BCG. Of the 19 vaccinated WTD in that study, six animals cultured positive for BCG. We collected tissues for culture between five and six months after vaccination in 12 of the 14 vaccinates; it is likely that the bacilli were no longer present or were at undetectable levels in these animals and that suspected transmission may have occurred much earlier. However, the two animals that died at five and 12 weeks post-vaccination were not culture positive either.

As in previous studies we were able to detect NTM species in both deer and cattle [8], [14]. Typically, NTM are found in approximately 5–10% of deer tissues submitted for mycobacterial culture at NVSL (unpublished data). *Mycobacterium avium hominisuis* is considered an opportunistic pathogen of humans and pigs and has been shown to produce lesions in both species, although we did not detect any lesions in the deer or cattle. *Mycobacterium setense* has only rarely been reported in human subjects and is largely considered an organism of opportunistic infections [15], [16], [17]. Non-tuberculous *Mycobacterium* spp., such as *M. avium* spp. and *M. kansasii*, can potentially act as confounders in testing cattle for *M. bovis* infection [18]. To reduce this chance of false positives, comparative cervical tests were performed in both species, which measures not only the animal’s reaction to *M. bovis* antigens, but also to *M. avium* spp. In this study, two control deer with NTM became CCT suspect and two became positive and it is therefore possible that these outcomes were influenced by the NTM infections.

Five orally vaccinated deer died prematurely in this study with no deaths in the non-vaccinated group. In a previous study four of the six deer that died prematurely were parenterally vaccinated with BCG [8]. While these numbers are too small to rule out random chance, the data are concerning enough for WTD to support further research on vaccine safety. Such future studies must include placebo groups to fully assess the safety of BCG and the safety of any delivery vehicles, such as baits.

In conclusion, transmission of BCG from orally vaccinated deer to cattle through a contaminated environment is probably of minimal concern to managers considering oral vaccination of free-ranging white-tailed deer in Michigan. As the sharing of feed and other resources between deer and cattle is one of the primary modes of disease transmission of virulent *M. bovis*, possible exposure of cattle to BCG was an important issue for investigation. However, these findings represent a small-scale attempt to answer the many relevant questions being asked by researchers tasked to evaluate the feasibility of using BCG in wild deer. More information is still greatly needed regarding safety of the vaccine in deer and non-target species, including domestic cattle.
